# Adeno-Associated Virus Gene Therapy: Is There a Risk of Insertional Mutagenesis?

**DOI:** 10.1177/01926233261448135

**Published:** 2026-05-31

**Authors:** Paul Batty

**Affiliations:** 1 University College London, London, UK; 2 Royal Free Hospital NHS Foundation Trust, London, UK

**Keywords:** gene therapy, adeno-associated virus, insertional mutagenesis, cancer

## Abstract

Adeno-associated virus (AAV) gene therapy has received approvals for the treatment of a number of rare monogenic disorders. There are questions surrounding the natural history of recombinant AAV (rAAV) vectors, which could provide insights into durability and long-term safety. Wild-type AAV (wtAAV) persists in extrachromosomal episomes and is also able to actively or passively integrate into the host genome. Although clonal wtAAV integration has been described in a small number of hepatocellular carcinoma cases, this at most represents a minor risk factor. Differences in the structure of wtAAV and rAAV limit translation of these studies. Recombinant AAV also persists in episomal forms with a small proportion becoming integrated. Some early murine studies demonstrated insertional mutagenesis after treatment with rAAV. These findings have not consistently been seen and are likely context-dependent (high-dose or neonatal treatment). There have been no reports of rAAV insertional mutagenesis in large animal models or human biopsy samples.

## Introduction

Adeno-associated virus (AAV) gene therapy has received licensing approvals for treatment of a number of rare monogenic disorders (Table 1). This approach uses recombinant viral vectors to deliver a functional copy of a gene to a target cell to allow long-term endogenous protein production. Although this approach has provided long-term therapeutic expression in clinical studies,^26^ there are questions surrounding the cellular mechanism(s) of vector persistence. Recombinant AAV (rAAV) vectors predominantly persist in extrachromosomal episomal (monomeric or concatemeric) forms with only rare integration events into the host genome.^1,28^ Although integration forms a minor part of rAAV persistence, understanding the mechanisms and locations of these events is important to inform on long-term efficacy and safety. This review will provide an update on recent advances evaluating the natural history of wild-type and rAAV and how these studies inform on whether there is a risk of insertional mutagenesis.

**Table 1.** Recombinant adeno-associated viral vectors approved and commercially available in 2025.

Vector (brand name)	Gene	Capsid	Indication	Route	FDA	EMA	NICE
Voretigene neparvovec **Luxturna**	*RPE65*	AAV2	Inherited retinal dystrophy (biallelic*RPE65* mutation)	Subretinal	2017	2018	2019
Onasemnogene abeparvovec **Zolgensma** **Itvisma**	*SMN1*	AAV9	Spinal muscular atrophy (SMA)	Intravenous Intrathecal	2019 2025	2020 NA	2021 NA
Etranacogene dezaparvovec **Hemgenix**	*F9*	AAV5	Severe & moderately severe hemophilia B (HB)	Intravenous	2022	2023	2024
Valoctocogene roxaparvovec **Roctavian**	*F8*	AAV5	Severe hemophilia A (HA)	Intravenous	2023	2022	NA
Eladocagene exuparvovec **Kebilidi/Upstaza**	*DDC*	AAV2	Aromatic l-amino acid decarboxylase (AADC) deficiency	Intraputaminal	2024	2022	2023
Delandistrogene moxeparvovec **Elevidys**	*DMD*	AAVrh74	Duchenne muscular dystrophy (DMD)	Intravenous	2023	NA	NA

Abbreviations: FDA = United States Food and Drug Administration; EMA = European Medicines Agency; NICE = National Institute for Health and Care Excellence, UK.

## Natural History of Wild-Type AAV

Wild-type adeno-associated virus (wtAAV) is a single-stranded DNA virus of the Parvoviridae family. The wtAAV structure consists of *Rep* and *Cap* sequences between two inverted terminal repeats (ITRs). These encode proteins required for replication, packaging, persistence, and capsid production. This is contained within a capsid structure, with 13 capsid serotypes identified. Population studies have demonstrated high seroprevalence of wtAAV antibodies with no clear link to pathological disorders. Wild-type adeno-associated virus is replication-deficient and requires a helper virus for active replication. In the absence of a helper virus, wtAAV persists in a latent form in extrachromosomal episomes or via integration into the host cell genome. Integration can occur passively or by site-directed insertion mediated by a *Rep*-encoded protein complex. This complex recognizes consensus Rep-binding sequences (RBS) in the host genome and viral ITR/promoter. This results in common genomic integration sites (ISs), with the first characterized site (AAVS1) located in exon 1 of the protein phosphatase 1 regulatory subunit 12C (*PPP1R12C*) gene on chromosome 19q13.42.^15^ Studies in HeLA cells confirmed the presence of AAVS1, albeit at lower frequency (10%) and identified other novel recurrent IS on chromosome 5p13.3 (AAVS2) and 3p24.3 (AAVS3). Alongside this, integration was seen across the genome with a bias towards open chromatin regions at consensus RBS.^12^ Studies in human diploid fibroblasts demonstrated the presence of AAVS1, with novel recurrent ISs on chromosome 1q25.3, 7q32.3, and 5q31.2, with only rare events at AAVS2 (n = 3) and AAVS3 (n = 1). Recurrent ISs occurred in vicinity to consensus RBS-like sequences with GAGY/C repeats and areas of open chromatin.^11^ Differences in integration patterns between these studies likely reflect differences in chromatin accessibility and proliferative capacity of these cell lines. These in vitro findings are supported by a study of liver samples from non-human primates (NHPs) and humans.^19^ Unique integration loci (UIL) of wtAAV were seen across the genome with enrichment in genomic regions highly expressed in the liver, open chromatin regions, and areas with increased susceptibility to DNA damage. Only small clonal populations of cells containing integration loci were seen. Integration was seen in AAVS1, AAVS2, and AAVS3 accounting for an average of 7.2% of UIL. In summary, in vitro and in vivo studies of wtAAV have demonstrated integration occurring via site-directed mechanisms and passively at regions of open chromatin or areas susceptible to DNA damage.

## Wild-Type AAV Integration: Is There a Risk of Insertional Mutagenesis?

With the identification of wtAAV integration, this raises the question as to whether these events have the potential to result in insertional mutagenesis. Concerns regarding this were first published in a French study that detected a 208-base pair (bp) insertion of AAV2 on screening hepatocellular carcinoma (HCC) samples for *TERT* promoter mutations.^20^ Analysis of these samples demonstrated clonal ISs of AAV2 in 5.7% (11/193) of HCC samples in *CCNA2* (n = 4), *CCNE1* (n = 3), *TERT* (n = 1), *TNFSF10* (n = 2), and *KMT2B* (n = 1).^20^ A common AAV2 region inserted included a region in the 3′UTR demonstrated to have enhancer/promoter activity.^17^ More detailed studies have been presented by this group in a larger cohort of 1461 patients (HCC = 936).^16^ In this study, AAV2 or AAV13 was detected in 21% of liver samples, with higher rates of positivity in non-tumor compared with tumor samples (18% v 8%).^16^ Clonal wtAAV integration was seen in 2% of HCC specimens, targeting *CCNA2* (33.3%), *CCNE1* (27.8%), *GLI1/INHBE* (11.1%), *TERT* (11.1%), *TNFSF10* (11.1%), and *KMT2B* (5.6%).

In contrast to these European studies, far lower incidence (0%-1.1%) of clonal wtAAV integration events has been reported in non-European cohorts.^7,22,30,33^ A study evaluating HCC samples in Japanese patients reported clonal AAV2 integration in 1.1% (3/268), with insertions in *KMT2B*, *CCNE1*, and an intergenic region of chromosome 5. All three patients had coexistent hepatitis B (HBV, n = 1) or hepatitis C (HCV, n = 2) infection.^7^ A second study from Nihon University School of Medicine investigated 243 liver and HCC samples demonstrating AAV2 ISs in three patients: two with prior HBV infection (*CCNE1* and *CCNA2*) and one patient without HBV or HCV (*SLC6A5*).^33^ Similarly, a study from Korea demonstrated AAV2 in 0.7% (2/289) HCC cases, with both patients having a history of HBV (n = 2) and/or alcohol intake (n = 1). Detailed evaluation of 117 HCC samples from Thailand and Mongolia demonstrated a single potentially causative AAV2/13 IS in *CCNA2* and concluded that these events are rare and present minimal evidence of contribution to oncogenesis in HCC. Finally, a large study evaluating multiple tumor types from West China Hospital of Sichuan University, demonstrated high rates of AAV positivity (80%) in both tumor and adjacent tissue; however, these lacked clonality.^24^ There are differences in the rates of reported clonal AAV2 IS in European and non-European populations. In studies that reported clonal integration, it is interesting to note that there is an overlap in targeted genes to those seen for HBV (eg, *CCNA2*, *CCNE1*, *TERT*, and *KMT2B*).

With high seroprevalence of AAV2 infection and low frequencies of clonal integration events in most studies, it is likely that wtAAV plays at most a very minor role in HCC oncogenesis. Due to significant differences between rAAV and wtAAV, these studies also provide limited information to inform on long-term safety of rAAV vectors. Recombinant AAV vectors contain no viral coding sequences, which removes the ability for site-directed integration. This has been seen in vitro, with loss of IS clusters when comparing *Rep*- and *Cap*-deficient rAAV vectors to wtAAV.^11^ Another important difference in the design of rAAV vectors is the removal of 3′ regions with promoter/enhancer activity,^17^ which are postulated as a mechanism driving rare clonal integration events for wtAAV.^30^

## Recombinant AAV Integration: Is There a Risk of Insertional Mutagenesis?

Preclinical and clinical studies evaluating the mechanisms of long-term persistence of rAAV have demonstrated that these vectors predominantly persist in extrachromosomal episomal forms with rare integration events.^2^ Although integration could provide one mechanism for stable long-term expression, this also raises the question on whether this could result in insertional mutagenesis. A small murine study conducted in 2001 first raised questions around whether rAAV might be associated with insertional mutagenesis.^6^ This study, which was not designed to assess toxicity, was conducted in mucopolysaccharidosis (MPS) type VII mice treated in the neonatal period with an AAV2-CAG-GUSB vector. On histology, six mice were found to have developed HCC and/or angiosarcoma at up to 18 months follow-up. Analysis by polymerase chain reaction (PCR) demonstrated low or undetectable AAV copy numbers, suggesting that it was unlikely these tumors resulted from clonal expansion, but required further study. A larger follow-up study demonstrated HCC formation in 33% to 56% of MPS type VII and wild-type mice.^5^ Recurrent ISs were detected in the murine *Rian* (RNA imprinted and accumulated in the nucleus) locus on chromosome 12, a region containing multiple small nucleolar RNA and micro-RNA genes. In contrast, a recent longitudinal study in wild-type mice demonstrated episomal/concatemeric forms made up 95% of all sequencing reads, with only low-frequency integration events (1.8-2.7 per 1000 cells) following treatment with an AAV5-hA1AT vector.^13^ Integration was polyclonal, with no evidence of clonal expansion or tumor formation at 57 weeks postdosing. Although common ISs were seen, only a single IS was seen in the *Rian* locus. Alongside this, one study has described the occurrence of a lung tumor in a Sandhoff disease mouse model. In this study, mice were treated with an AAV9-HexB construct in the neonatal period and followed up for 43 weeks. Liver tumors were seen in 7/10 mice, reported as showing benign hyperplasia but could not rule out hepatic adenoma, with integration mapping to the *Rian* locus. In one mouse, a lung tumor was detected with integration in the fibroblast growth factor 2 receptor (FGFR2). In this study, however, clonality was not evaluated to determine whether these integration events could be drivers for insertional mutagenesis.^3,34^ There have been conflicting findings in studies evaluating whether there is a risk of insertional mutagenesis in rodent models. Where this has been described, this is likely to be context-dependent, associated with higher vector doses, neonatal treatment, and some vector promoter/enhancer elements.^29^ Finally, a recent study has highlighted that host factors may influence the incidence of tumor formation in mice treated with rAAV.^4^ In this study, adult mice treated with a *Rian* targeting rAAV vector did not develop HCC unless fed with a high-fat diet, leading to inflammation and hepatocyte proliferation. Interestingly, lower incidence of HCC was seen in female mice or male mice treated with estrogen. Further investigation is required in this area, as these findings may have translational relevance with the rising incidence of metabolic dysfunction–associated steatotic liver disease.

In large animal models and clinical studies, long-term efficacy has been reported for rAAV with no evidence of tumorigenesis.^2^ Evaluation of integration in biopsy samples from these studies has provided insights into the natural history of rAAV. In two long-term follow-up studies conducted in the hemophilia A dog model, rAAV integration was seen, with overlapping common ISs in proximity to *EGR2*/*MIR1296*, *ALB*, *CCND1*, and *EGR3*/*MIR320*.^1,21^ In the first study, ISs were enriched within transcriptional units, with a modest increase in integration event relative to known cancer genes.^21^ During follow-up in this study, increased transgene expression was seen over time in two dogs, which was described to have resulted from clonal expansion of cells containing integrated rAAV. Of note, the construct used included the wtAAV 3′ promoter/enhancer sequence which may have contributed to this process. The second study described predominant episomal vector persistence, with average integration frequencies of 9.3e-4 events/cell. There was enrichment of integration events in areas of open chromatin, with no increase in integrations sites proximal to known protooncogenes.^1^ In both canine studies, there was no evidence of tumorigenesis. There have been two recent reports evaluating rAAV integration in NHP.^9,19^ In the first of these studies, integration was seen across the genome with enrichment in regions highly expressed in the liver, open chromatin regions, and areas with increased susceptibility to DNA damage.^19^ There was no difference in number of ISs relating to age at treatment (adult vs neonatal), transgene, promoters, or follow-up timepoint. In the second study, integration data are presented from NHPs treated with AAV8 and AAVrh10 constructs expressing β-choriogonadotropic hormone (rh-β-CG; CGB), human or macaque low-density lipoprotein receptor, or green fluorescent protein.^9^ At day 182 for NHP treated with the rh-β-CG construct, integration frequencies were in the range of 0.1 to 1.6 events/100 genomes. Integration was seen across the genome, with enrichment in and around genes highly expressed in the liver. There was no evidence of marked clonal expansion or integration in regions mutated in human HCC.

There are limited studies evaluating the natural history of rAAV in humans post gene therapy. Liver biopsy samples have been evaluated after rAAV treatment for acute intermittent porphyria (n = 3)^8^ and hemophilia A (n = 5).^28^ In the first study, after 1 year of follow-up, integration frequencies of 1.17e-3 events/cell were reported, with no recurrent ISs and no integration in regions associated with HCC.^8^ In liver biopsies obtained 0.5 to 4.1 years post AAV5-hFVIII-SQ treatment in patients with severe hemophilia A, integration was seen across the genome with average frequencies of 3.97e-3 events/cell. There was no increase in integration events proximal to cancer genes and no evidence of clonal expansion.

## Investigation of Malignancies in Gene Therapy Recipients

Within clinical gene therapy studies, pathways have been developed to investigate malignancies to assess for genotoxicity. These require different tissue sampling pathways than are used in routine clinical practice for histopathological diagnosis. This requires preservation of tissue biopsies in formalin for histological studies and flash freezing for molecular studies. Integration studies benefit from availability of surrounding non-tumor tissue to provide context. The first stage in the molecular diagnostic pathway involves assays to detect and quantify rAAV, for example, quantitative PCR and in situ hybridization. Of note, these assays do not differentiate between episomal or integrated forms and could underestimate vector copy numbers if rAAV fragments are present. Integration site analysis is used to identify junctional sequencing reads that contain both vector and host genome sequences. Integration site analysis can be untargeted, for example, whole genome sequencing or targeted (PCR-based or non-PCR-based) to enrich IS junctions, with both being coupled to next-generation sequencing. Polymerase chain reaction–based assays (eg, linear amplification or ligation-mediated PCR) use single or multiple probe sets targeting the ITR and/or other rAAV regions. Non-PCR-based approaches (eg, target enrichment sequencing) use overlapping oligonucleotide capture baits that cover the whole vector sequence. This approach has the potential advantage of being able to capture fragmented vector sequences. Integration site analysis provides information on the location and frequency of ISs, with downstream bioinformatic analyses being required to evaluate for insertional mutagenesis or clonal expansion. Analysis of tumor samples will often include evaluation for tumor-specific (non-AAV) somatic mutations or expression profiles. A recent study elegantly outlines this pathway used to investigate a case of HCC in a man with moderately severe hemophilia B treated with an AAV2/5-LP1-hFIXco-R338L vector.^31^

There have been eleven cases of cancer reported in clinical trial participants treated with rAAV vectors.^10,14,18,23,25-27,31,31^ These events have occurred after variable follow-up (<1 to >11 years) and have included both solid and liquid tumors (Table 2). A recent update from REGENXBIO has reported an intraventricular central nervous syndrome tumor detected on routine imaging in a study participant four years after treatment for mucopolysaccharidosis type I (MPS I) with intracisternal AAV9-IUDA vector.^25^ Preliminary data from the resected tumor have reported an AAV vector genome integration event associated with overexpression of pleomorphic adenoma gene 1 (PLAG1). Within this report, it is stated that causality has not been established. Further details of the molecular studies are awaited to establish whether this could represent AAV-driven insertional mutagenesis. For all other cases, detailed molecular analyses have provided no evidence of insertional mutagenesis.

**Table 2.** Investigation of malignancies in clinical trial participants demonstrates no evidence of insertional mutagenesis.

Vector	Dx	Age, y	Sex	Timing, y	Tumor	Summary of molecular testing	References
AAV8.sc-TTR-FIXR338Lopt **BAX335**	HB	69	M	NA	Squamous cell carcinoma of the tonsil	No evidence of AAV VG and/or integration into the tonsillar tumor tissue	14
AAV2/5-LP1-hFIXco-R338L **Etranacogene dezaparvovec**	HB	69	M	1	Hepatocellular carcinoma	Similar VCN (copies/cell) in tumor (3.21) vs normal tissue (4.11). Low integration frequency: 1 in 10,000 cells. No evidence of clonal expansion. WGS: non-AAV-related Chr 8 deletion & mutations in *TP53*, *NFE2L2*, and *PTPRK.*	31
		*NA*	*M*	*NA*	*Schwannoma*	*Unrelated to treatment on molecular analysis*	*23* ^a^
		*NA*	*M*	*NA*	*Myelodysplastic syndrome*	*Unrelated to treatment on molecular analysis*	*23* ^a^
ssAAV2/5-HLP-hFVIII-SQ **Valoctocogene roxaparvovec**	HA	47	M	>5	Parotid acinic cell carcinoma	Similar integration frequency in tumor vs healthy tissue: 6.5e−5 vs 1.0e−4 IS/cell. No evidence of clonal expansion. Polyclonal integration pattern.	32
		20	M	3	B-cell acute lymphoblastic leukemia (B-ALL)	ddPCR: Low AAV VCN <1 copy/100 cells. No AAV integration sites. Known ALL mutational driver (AAV-unrelated) in 85% of BM cells: CRLF2 rearrangement.	18
scAAV2/8-LP1-hFIXco	HB	74	M	11.6	Prostate adenocarcinoma	ddPCR: AAV not detected. Likely to be unrelated.	26
		44	M	7	Non-mucinous lung adenocarcinoma in situ	ddPCR: Similar VCN in tumor and surrounding tissue (0.04 vs 0.05 copies/RNaseP). Insufficient tissue for further testing. Likely to be unrelated.	26
scAAV2/9-CMV_en_CB-SMN1 **Onasemnogene abeparvovec**	SMA	1.3	M	1.2	Epithelioid neoplasm of the spinal cord	ISH: Vector (total) copies detected in many, but not all tumor cells. ISA failed to detect high confidence IS due to limited tissue: Five unique IS in 3 samples.	27
		2	M	0.67	Pilocytic astrocytoma	ddPCR: 0.7-4.9 copies/dg. ISH: Variable transduction in individual tumor cells. Low total number of IS (n = 71). No integration proximal to cancer genes resulting in clonal expansion.	10
AAV9-CB7-cohIDUA RGX-111	*MPS I*	*5*	*NA*	*4*	*Intraventricular CNS tumor*	*Vector integration event associated with overexpression of pleomorphic adenoma gene 1 (PLAG1). Causality not established. Ongoing investigations awaited.*	25^a^

Abbreviations: AAV = adeno-associated virus; BM = bone marrow; CNS = central nervous system; CRLF2 = cytokine receptor–like factor 2; ddPCR = droplet digital polymerase chain reaction; Dx = diagnosis; HA = hemophilia A; HB = hemophilia A; ISs = integration sites; ISA = integration site analysis; ISH = in situ hybridization; M = male; MPS = mucopolysaccharidosis; NA = not available; SMA = spinal muscular atrophy; VCN = vector copy number; VG = vector genomes; WGS = whole genome sequencing; y, years.

a Presented as an abstract or press release only.

## Conclusion

Recombinant AAV vectors provide a potentially transformative treatment option for individuals with rare monogenic disorders. Natural history studies have demonstrated that these vectors predominantly persist in episomal forms, with low frequencies of integration into the host genome. Although early murine studies reported concerns of insertional mutagenesis for rAAV, these events were likely context-dependent and associated with high vector doses or treatment in the neonatal period. There have been no reports of insertional mutagenesis in large animal models or clinical studies. There is a need for ongoing registry and real-world studies to improve understanding of long-term efficacy and safety of rAAV gene therapy.
